# Associations between Folate and Vitamin B12 Levels and Inflammatory Bowel Disease: A Meta-Analysis

**DOI:** 10.3390/nu9040382

**Published:** 2017-04-13

**Authors:** Yun Pan, Ya Liu, Haizhuo Guo, Majid Sakhi Jabir, Xuanchen Liu, Weiwei Cui, Dong Li

**Affiliations:** 1 Department of Nutrition and Food Hygiene, School of Public Health, Jilin University, 1163 Xinmin Avenue, Changchun 130021, China; yunpan14@mails.jlu.edu.cn (Y.P.); liuya@jlu.edu.cn (Y.L.); liuxc15@mails.jlu.edu.cn (X.L.); 2 Department of Radiology, The Second Part of the First Hospital, Jilin University, Changchun 130031, China; doyouknow_0330@163.com; 3 Department of Biotechnology, University of Technology, Baghdad 00964, Iraq; msj_iraq@yahoo.com; 4 Department of Immunology, College of Basic Medical Sciences, Jilin University, 126 Xinmin Avenue, Changchun 130021, China; 5 Department of Hepatology, The First Hospital, Jilin University, Changchun 130021, China

**Keywords:** folate, vitamin B12, inflammatory bowel disease, meta-analysis, nutrition

## Abstract

Background: Inflammatory bowel disease (IBD) patients may be at risk of vitamin B12 and folate insufficiencies, as these micronutrients are absorbed in the small intestine, which is affected by IBD. However, a consensus has not been reached on the association between IBD and serum folate and vitamin B12 concentrations. Methods: In this study, a comprehensive search of multiple databases was performed to identify studies focused on the association between IBD and serum folate and vitamin B12 concentrations. Studies that compared serum folate and vitamin B12 concentrations between IBD and control patients were selected for inclusion in the meta-analysis. Results: The main outcome was the mean difference in serum folate and vitamin B12 concentrations between IBD and control patients. Our findings indicated that the average serum folate concentration in IBD patients was significantly lower than that in control patients, whereas the mean serum vitamin B12 concentration did not differ between IBD patients and controls. In addition, the average serum folate concentration in patients with ulcerative colitis (UC) but not Crohn’s disease (CD) was significantly lower than that in controls. This meta-analysis identified a significant relationship between low serum folate concentration and IBD. Conclusions: Our findings suggest IBD may be linked with folate deficiency, although the results do not indicate causation. Thus, providing supplements of folate and vitamin B12 to IBD patients may improve their nutritional status and prevent other diseases.

## 1. Introduction

Inflammatory bowel disease (IBD) is characterized by chronic and typically recurrent intestinal inflammation, and it includes Crohn’s disease (CD) and ulcerative colitis (UC). Although the exact aetiology and pathogenesis of IBD is still largely unknown, it is considered to be related to individual immunity, an inherited predisposition, environmental factors and the interactions between the mucosal immune system and intestinal antigenic material (e.g., commensal bacteria) [1]. Abnormal immune responses are believed to be the direct cause of intestinal damage [2]. IBD can lead to many clinical symptoms, including impaired nutrient absorption, which can influence the absorption of folate and vitamin B12. Furthermore, many studies have indicated that serum folate and vitamin B12 concentrations influence the development of IBD. Folate is involved in the methylation of DNA and may produce epigenetic changes that affect the interaction between the gut microbiota and systemic immune responses [3]. The gut microbiota [4] and epigenetic changes [5] may be involved in the pathogenesis of IBD. Vitamin B12 acts as a coenzyme in various biochemical reactions, including DNA synthesis and folate metabolism [6]. Deficiencies in vitamin B12 and folate can lead to macrocytic anaemia, hyperhomocysteinemia, and neurologic and psychiatric disorders [7,8,9]. Compared with healthy subjects, IBD patients are at increased risk of hyperhomocysteinemia [10], and folic acid and vitamin B12 may play pivotal roles in homocysteine metabolic reactions [11]. Moreover, folate and vitamin B12 deficiencies may cause increased homocysteine levels, a risk factor for thrombosis [8,12,13,14]. 

Many studies have reported that serum folic and vitamin B12 concentrations differ between IBD patients and healthy individuals. However, the results are not consistent, and differences have been observed between patients with CD and UC. Whether serum folic acid and vitamin B12 concentrations are lower in IBD patients than in non-IBD patients is still largely unknown. Thus, a more comprehensive evaluation of the association between IBD and serum folic acid and vitamin B12 concentrations is needed. In this study, we conducted a meta-analysis to analyse the relationships between the serum concentrations of folic acid and vitamin B12 in IBD patients and healthy controls to provide additional insights into treating and rehabilitating IBD and maintaining a healthy nutritional status. 

## 2. Materials and Methods

### 2.1. Sources and Methods of Data Retrieval

We performed a comprehensive literature search that included studies from 1970 to December 2016; the electronic databases included PubMed, Medline, Web of Science, and Google Scholar. The searches were conducted to identify all published studies that reported data on the mean differences and standard deviations of serum folate and vitamin B12 concentrations in IBD patients and healthy controls. The following terms were used for the literature search: folic acid, vitamin B9, vitamin M, folvite, folate, vitamin B12, cyanocobalamin, cobalamins, inflammatory bowel disease, Crohn’s disease, ulcerative colitis. The term ‘OR’ was used as the set operator to combine different sets of results. The serum concentrations of folate and vitamin B12 and IBD were determined and used in a meta-analysis to understand how serum folate and vitamin B12 concentrations differ in IBD patients relative to healthy controls. Age, location, detection methods and other confounding factors were also considered.

### 2.2. Inclusion Criteria

**Figure 1:**
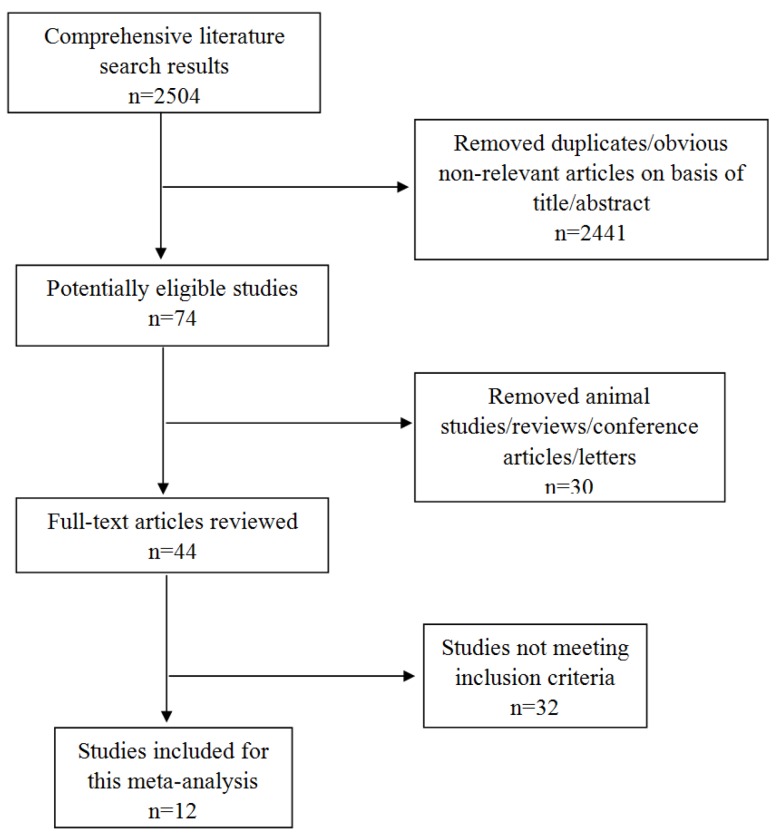
Flow diagram of the literature search.

The articles that were included in this meta-analysis matched the following five criteria: (1) inflammatory bowel disease patients were clinically diagnosed; (2) studies included a case group and a control group; (3) the folate and vitamin B12 values were presented as the mean ± standard deviation (SD); and (4) the patients and controls had not previously received folate and vitamin B12 supplementation; and (5) we excluded studies that did not provide initial data, animal studies, in vitro studies, reviews and conference papers. Three investigators independently reviewed and extracted all of the potentially eligible studies and discussed the inconsistencies until a consensus was reached (Figure 1). Additionally, the Newcastle-Ottawa Scale was used for assessing the quality of studies included in this meta-analysis (Table 1).

### 2.3. Data Abstraction

We reviewed all of the relevant studies and extracted the following data: (1) lead author, publication year, sample size, mean age of the patients and controls, and gender of the patients and controls; (2) serum folate and vitamin B12 concentrations of the patients and controls; (3) folate and vitamin B12 detection methods; and (4) the diagnosis of the patients and the number of patients and controls.

### 2.4. Statistical Analysis

All statistical analysis was conducted using the statistical software Stata (version 12.0, StataCorp LLC, College Station, TX, USA). The mean difference, standard deviation and standard error of the serum folate and vitamin B12 concentrations in the IBD and control group were used for the meta-analysis. Units that were not unified were transformed into unified units. We combined the standardized mean difference (SMD) for studies that reported mean and standard deviation values for serum folate and vitamin B12 concentrations in IBD patients and controls. An inverse variance weighted random effect model was used to determine the SMD and 95% confidence intervals (CIs) and measure the different concentrations of folate and vitamin B12 in the patients and controls, and the results were used to evaluate the differences in serum concentrations of folate and vitamin B12 between the IBD patients and normal controls. In order to avoid double counting, both controls in two studies that included both UC and CD patients [15,16] were split approximately evenly into 2 control groups with the means and standard deviations left unchanged before entered into the meta-analysis [17,18]. 

We used Cochran’s Q statistic and the *I*^2^ statistic to assess the statistical heterogeneity in the meta-analysis [19]. If the data were homogeneous (*p* > 0.05), a fixed effect model meta-analysis was performed; if the data were heterogeneous (*p* ≤ 0.05), a random effects model meta-analysis was performed. Heterogeneity was considered significant at *p* < 0.05 in the Q test, and the *I*^2^ value was used to evaluate the degree of heterogeneity. We defined low, medium and high heterogeneity at *I*^2^ values of 25%, 50%, and 75%, respectively [20]. Sensitivity analysis was used for analyzing heterogeneity. Subgroup analyses were performed for the type of disease, region of study, and method of detecting the vitamins. In addition, we performed a meta-regression analysis based on the folate and vitamin B12 measurement methods, the year of publication, the sample size, the quality of the study and the average age of the patients. We used a funnel plot to detect publication bias concerning this meta-analysis, with the symmetry of the funnel plot used to determine whether publication bias occurred. Furthermore, a formal statistical assessment of the funnel plot asymmetry was performed with Egger’s regression asymmetry test [21].

## 3. Results

Our search identified 2504 related references; however, only 12 papers met our inclusion criteria. The 12 studies included 2570 individuals in total, with 1086 IBD patients and 1484 controls [15,16,22,23,24,25,26,27,28,29,30,31]. The detailed results are expressed in Table 1. Among the 12 studies, the folate concentration in the study by Kuroki et al. [24] was excluded because it was detected in red blood cells. Folate and vitamin B12 were detected via chemiluminescence immunoassays in three studies [15,22,28], radioimmunoassays in four studies [23,24,25,30], ELISA in two studies [26,31], a specific immunochemical method in one study [29], and the IMx assay in one study [16]. Three studies did not include the mean age of patients and controls [15,16,23], and one study included the median age of patients and controls [23]. Of the 12 included studies, six studies were conducted in Asia [15,22,24,26,28,31], four in Europe [16,23,25,30], one in America [27], and one in Africa [29]. Seven studies investigated the association between UC and serum folate and vitamin B12 concentrations [15,16,22,27,28,30,31]; six studies investigated the association between CD and serum folate and vitamin B12 [15,16,23,27,29,30]; and two studies investigated IBD patients who were not classified by the type of disease [25,26]. The patients’ basic characteristics are presented in Table 1. The participants in one study [27] were children, and those in the other studies were adults. The data included in this analysis was defined as continuous variables.

**Figure 2:**
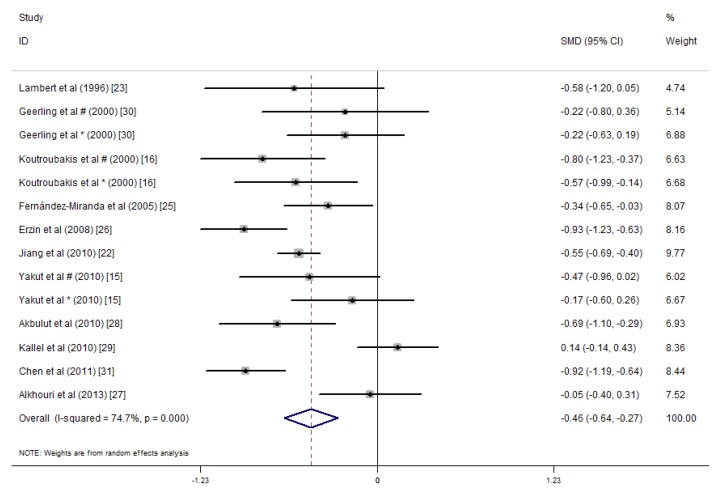
Forest plot of the serum folate concentrations in the inflammatory bowel disease (IBD) patients vs. controls; standardized mean differences with the 95% confidence interval and weight percentage are shown. * Reported in ulcerative colitis; ^#^ reported in Crohn’s disease.

**Figure 3:**
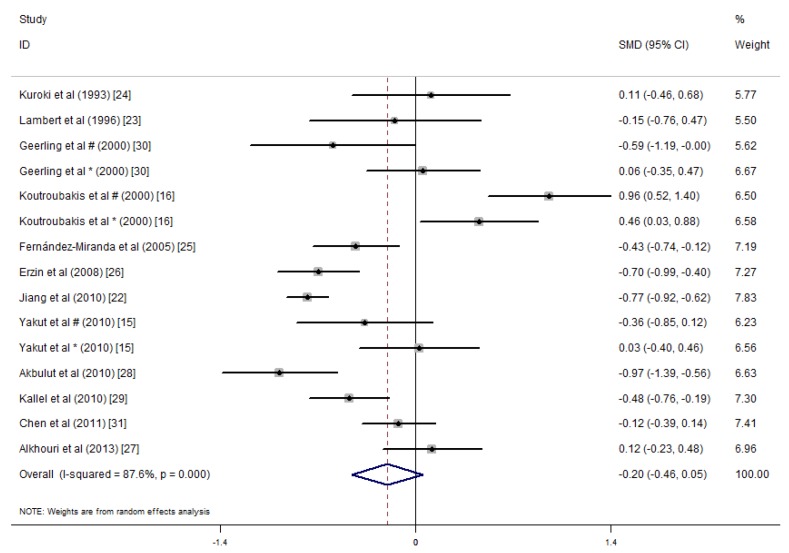
Forest plot of the serum concentrations of vitamin B12 in the IBD patients vs. controls; standardized mean differences with the 95% confidence interval and weight percentage are shown. * Reported in ulcerative colitis; ^#^ reported in Crohn’s disease.

We conducted a meta-analysis of the serum folic acid and vitamin B12 concentrations in 1086 IBD patients and 1484 controls. The average serum folate concentration in the IBD patients was 0.46 nmol/L lower than that in the controls (SMD = −0.46 ng/mL, 95% CI = −0.64, −0.27 ng/mL, *I*^2^ = 74.7%, *p* = 0.000; Figure 2). An analysis of the results of these studies indicated that IBD patients did not have significantly lower serum vitamin B12 concentrations than the healthy controls (SMD = −0.20 ng/mL, 95% CI = −0.46, 0.05 ng/mL, *I*^2^ = 87.6%, *p* = 0.123; Figure 3). Because of the heterogeneity of the results, we applied a random effects model. In addition, publication bias was not observed in the serum folate concentrations (Egger’s test: coefficient = 0.70, *p* = 0.627). However, publication bias was observed in the serum vitamin B12 concentration (Egger’s test: coefficient = 4.09, *p* = 0.021). A sensitivity analysis revealed that the studies by Koutroubakis et al. [16] influenced the results; however, when we excluded the study by Koutroubakis et al. on vitamin B12, our final results indicated that the serum vitamin B12 concentrations for IBD patients were 0.35 ng/mL lower than the controls (overall effect size = −0.35 ng/mL, 95% CI = −0.56, −0.13, *I*^2^ = 79.3%, *p* = 0.001).

Significant heterogeneity was observed for the studies (folate: *I*^2^ = 74.7%, *p* < 0.001; vitamin B12: *I*^2^ = 87.6%, *p* < 0.001). 

**Figure 4:**
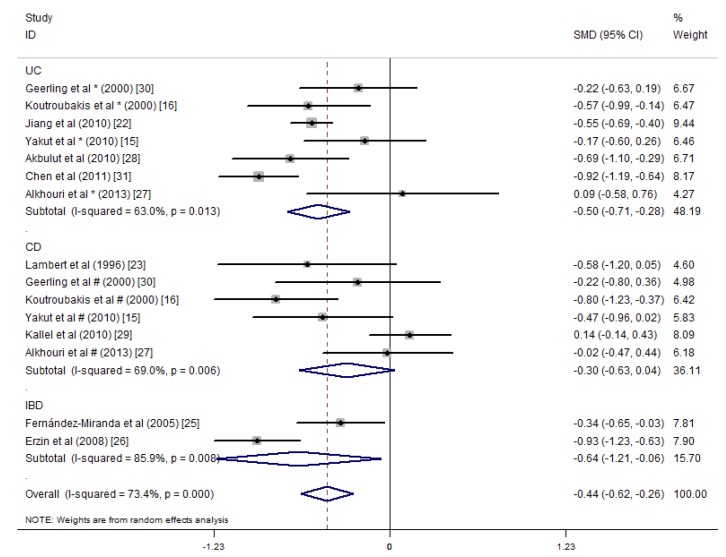
Forest plot of the serum concentrations of folate in the ulcerative colitis (UC), Crohn’s disease (CD) and IBD patients vs. controls; standardized mean differences with the 95% confidence interval and weight percentage are shown. Subtotals are for the UC, CD and IBD patients.

The included articles were divided into three groups by the type of disease as follows: only patients with UC (UC group) [15,16,22,27,28,30,31], only patients with CD (CD group) [15,16,23,27,29,30] and patients from studies [25,26] that did not indicate the type of IBD (IBD group). For the UC group (680 UC patients, 1151 controls), the average serum folate concentration was 0.50 ng/mL lower than that of the controls (SMD = −0.50 ng/mL, 95% CI = −0.71, −0.28 ng/mL, *I*^2^ = 63.0%, *p* = 0.000). For the CD group (247 CD patients, 367 controls), the average serum folate concentration was not statistically significant differences with the controls (SMD = −0.30 ng/mL, 95% CI = −0.63, 0.04 ng/mL, *I*^2^ = 69.0%, *p* = 0.080). For the IBD group, statistically significant differences with the controls were observed (SMD = −0.64 ng/mL, 95% CI = −1.21, −0.06 ng/mL, *I*^2^ = 85.9%, *p* = 0.030) (Figure 4).

**Figure 5:**
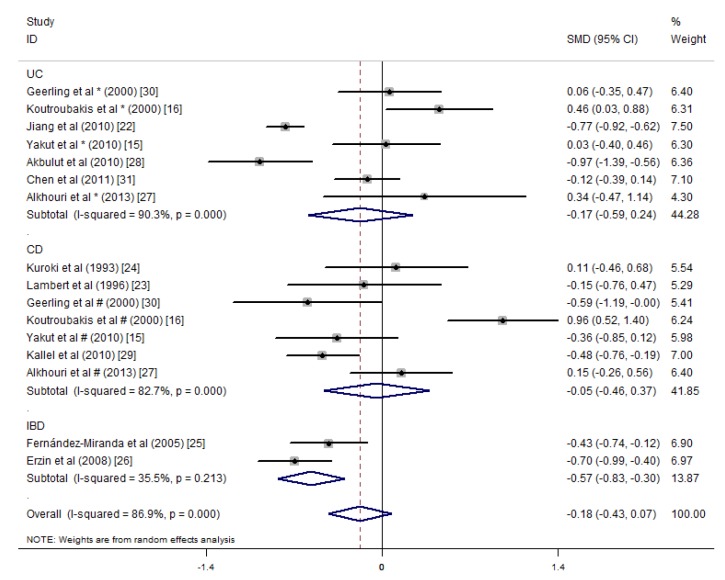
Forest plot of the serum concentrations of vitamin B12 in the UC, CD and IBD patients vs. controls; standardized mean differences with the 95% confidence interval and weight percentage are shown. Subtotals are for the UC, CD and IBD patients.

The meta-analysis of 12 studies that included the mean serum vitamin B12 concentrations showed an overall non-significant summary effect between the UC patients (SMD = −0.17 pg/mL, 95% CI = −0.59, 0.24 pg/mL, *I*^2^ = 90.3%, *p* = 0.406) and CD patients (SMD = −0.05 pg/mL, 95% CI = −0.46, 0.37 pg/mL, *I*^2^ = 82.7%, *p* = 0.829). However, IBD patients had significantly lower concentrations than healthy controls (SMD = −0.57 pg/mL, 95% CI = −0.83, −0.30 pg/mL, *I*^2^ = 35.5%, *p* = 0.000) (Figure 5).

**Figure 6:**
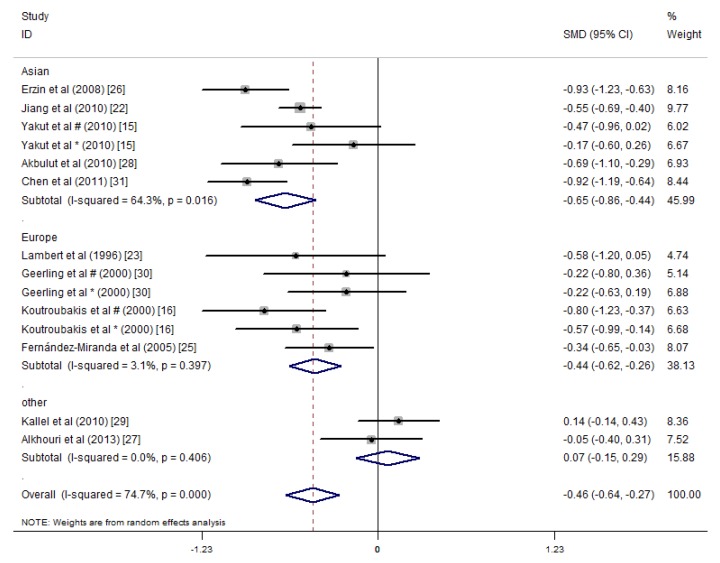
Forest plot of the serum concentrations of folate in the IBD patients vs. controls; standardized mean differences with the 95% confidence interval and weight percentage are shown. Subtotals are for the studies from Asia, Europe, and others. * Reported in ulcerative colitis; ^#^ reported in Crohn’s disease.

A subgroup analysis was performed according to the study area, and the studies were divided into three areas: Asia, Europe, and other (America and Africa). The studies in Asia showed that the serum folate concentrations of the IBD patients were significantly lower than those of the healthy controls (SMD = −0.65 ng/mL, 95% CI = −0.86, −0.44 ng/mL, *I*^2^ = 64.3%, *p* = 0.000). The studies in Europe showed that the serum folate concentrations in the IBD patients were 0.44 ng/mL lower than those of the healthy controls (SMD = −0.44 ng/mL, 95% CI = −0.62, −0.26 ng/mL, *I*^2^ = 3.1%, *p* = 0.000). The studies in the other areas did not show differences in the serum folate concentrations between the IBD patients and the controls (Figure 6).

**Figure 7:**
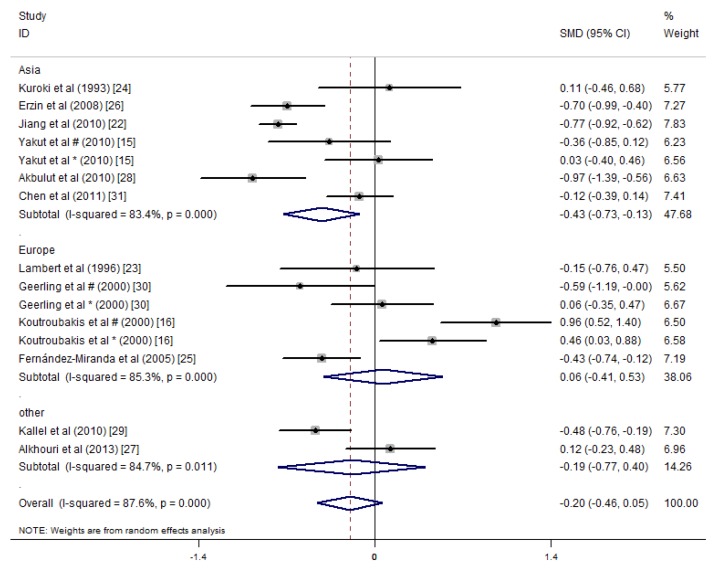
Forest plot of the serum concentrations of vitamin B12 in the IBD patients vs. controls; standardized mean differences with the 95% confidence interval and weight percentage are shown. Subtotals are for the studies from Asia, Europe, and others. * Reported in ulcerative colitis; ^#^ reported in Crohn’s disease.

In the subgroup meta-analysis on the mean serum vitamin B12 concentrations, the studies in Asia showed a significant summary effect, and the serum vitamin B12 concentrations of the IBD patients were 0.43 pg/mL lower than that of the controls (SMD = −0.43 ng/mL, 95% CI = −0.73, −0.13 ng/mL, *I*^2^ = 83.4%, *p* = 0.005). However, significant differences in serum vitamin B12 concentrations were not detected between the IBD patients and controls in the studies from Europe or the other areas (Figure 7). 

**Figure 8:**
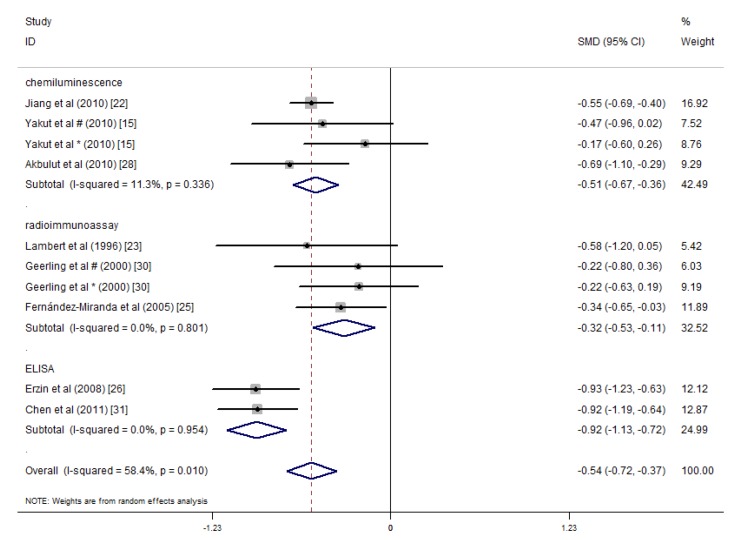
Forest plot of the serum concentrations of folate in the IBD patients vs. controls; standardized mean differences with a 95% confidence interval and weight percentage are shown. Subtotals are for the three detection methods. * Reported in ulcerative colitis; ^#^ reported in Crohn’s disease.

**Figure 9:**
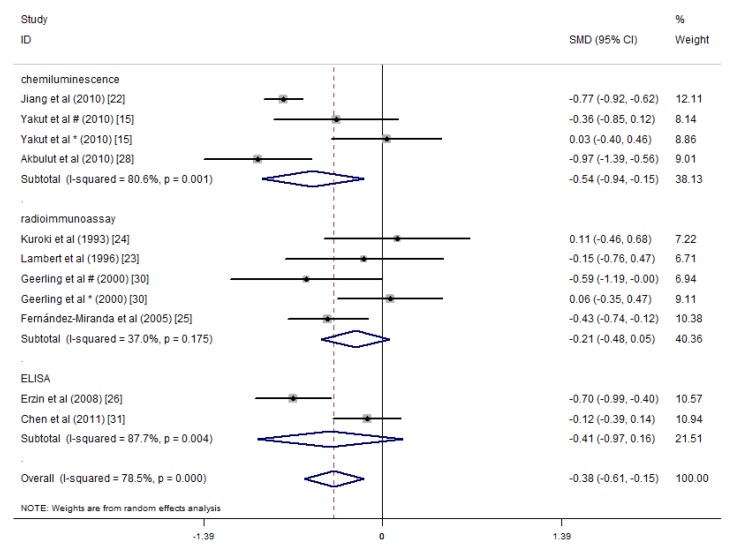
Forest plot of the serum concentrations of vitamin B12 in IBD patients vs. controls; standardized mean differences with a 95% confidence interval and weight percentage are shown. Subtotals are for the three detection methods. * Reported in ulcerative colitis; ^#^ reported in Crohn’s disease.

The main methods of detecting folic acid and vitamin B12 in the included studies were chemiluminescence immunoassays, radioimmunoassays and ELISA. Alkhouri et al. did not specify the detection method. Kallel et al.’s study used specific immunochemical methods [29], and Koutroubakis et al.’s study used the IMx assay [16]. We performed subgroup analysis according to the three main methods of detecting folate, and the results indicated that the heterogeneity among the three groups was reduced, and the subtotal *I*^2^ values of the chemiluminescence immunoassay, radioimmunoassay and ELISA methods were 11.3%, 0% and 0%, respectively. Studies that applied these three detection methods showed lower average serum folate concentrations in the IBD patients relative to the controls (Figure 8). Thus, the detection methods may introduce heterogeneity into the serum folate concentration results in the different studies. A similar subgroup analysis was performed for the serum vitamin B12 concentrations. Significant differences were only observed among the studies that used chemiluminescence immunoassays, and they indicated that the serum vitamin B12 concentrations of the IBD patients were 0.54 pg/mL lower than that of the controls (SMD = −0.54 pg/mL, 95% CI = −0.94, −0.15 pg/mL, *I*^2^ = 80.6%, *p* = 0.010). However, in studies that detected serum vitamin B12 concentrations via radioimmunoassays and ELISA, statistically significant differences were not observed between the IBD patients and the controls (Figure 9). 

A meta-regression analysis was performed because of the significant heterogeneity observed between studies. The meta-regression analysis evaluated the folate and vitamin B12 detection methods, the year of publication, the size of the samples, the quality of the study and the average age of the patients. The results showed that the detection methods impacted the serum folic acid concentrations and the total effect size (*p* < 0.05; Table 2). For serum vitamin B12 concentrations, the mean age of the IBD patients was the source of heterogeneity, and the difference in the results was statistically significant (*p* < 0.05; Table 3). The meta-regression analysis based on the size of the sample and the quality of study could not explain the between-study heterogeneity (Table 2 and Table 3).

## 4. Discussion

The results from this meta-analysis showed that the serum folate concentrations of patients with IBD were lower than that in normal controls, and the difference was significant. However, significant differences in serum vitamin B12 concentrations were not observed between the IBD patients and healthy controls. Serum folate concentrations may be reduced because of inadequate dietary intake [32], increased utilization, or drug effects, mainly salicylazosulfapyridine (SASP) [14]. Moreover, Burr et al. [33] showed that folic acid can be used as a supplement for the prevention of colorectal cancer in IBD patients. Considering the results of this study, IBD patients should be supplemented with folic acid. Research has shown that folate deficiency is more common than vitamin B12 deficiency [34]. Folate and vitamin B12 are important water-soluble vitamins for humans. Folate mainly assimilates in the duodenum and the proximal jejunum and is found in a variety of foods. Vitamin B12 is a component of coenzymes, and it is essential for cell biosynthesis and metabolism in vivo. Vitamin B12 is mainly absorbed in the terminal ileum, and the main source of vitamin B12 for humans is animal products. IBD may involve the small intestine; thus, the serum concentrations of folate and vitamin B12 may be lower in patients with IBD despite their presence in a wide range of foods. 

Although our findings indicated that the serum concentrations of vitamin B12 were not significantly different between IBD patients and controls, studies have shown that a resection of more than 50–60 cm of the ileum frequently produces vitamin B12 malabsorption [35]. The reason for this difference might be due to 50 patients among 1086 patients included in this meta-analysis underwent surgery, which is less than 5 percent. In CD patients, prior intestinal surgery was an independent risk factor for low serum concentrations of vitamin B12 [15]. A meta-analysis by Battat et al. on vitamin B12 deficiency in IBD patients indicated that the only factor that predisposed CD patients to vitamin B12 deficiency was ileal resections greater than 20 cm [36]. Therefore, IBD patients, especially patients who have had ileal surgery, are recommended to take vitamin B12 supplements.

In the subgroup analysis based on the types of diseases, the serum folate concentrations in UC patients but not CD patients were lower than those in the healthy controls. However, serum folate concentrations in patients with unspecified IBD were lower than that in controls. The subgroup analysis by area showed lower serum concentrations of folate and vitamin B12 in the Asian study populations, which may be related to the typical Asian diet, which mostly includes plant-based foods and less meat than is typical in European diets, because vitamin B12 widely occurs in animal-based food products. Therefore, we suggest these patients may require moderate vitamin B12 supplements.

When we combined the results of all of the studies, a large degree of heterogeneity was observed, so we performed analysis to identify the source of heterogeneity. Firstly, the subgroup analysis of the detection methods showed that the serum folate concentrations were lower in the IBD patients than in the healthy control patients, regardless of the techniques that were used (chemiluminescence immunoassay, radioimmunoassay or ELISA), and the heterogeneity was lower when grouping by detection methods compare to by disease types. Therefore, we concluded that different test methods for folic acid may be one of the sources of heterogeneity in the associated studies. Secondly, the results of the sensitivity analysis showed that the studies by Koutroubakis et al. introduced a large amount of heterogeneity [16]. This might be due to inclusion of three control groups (healthy blood donors, visitors to the gynaecology/obstetrics and orthopaedics wards and normal hospital personnel) by investigators (Koutroubakis et al. [16]), which might have introduced a number of uncontrollable confounding factors. Thirdly, the results of meta-regression analysis showed that the method of detection explained the heterogeneity among the studies that examined serum folate concentrations; the average age of the patient was a heterogeneous factor among the studies that examined the serum vitamin B12 concentrations. Moreover, IBD patients often present morphological and functional disorders of the liver [23], and patients with hepatic dysfunction may have normal or high serum concentrations of vitamin B12 rather than increased body stores of vitamin B12 [37]. We did not analyse the disease severity because of the different ratings criteria for severity. In addition, certain studies did not specify or excluded severe patients, which led to incomplete data; also, disease severity may be not associated with lower serum vitamin concentrations [24]. 

We still need to acknowledge that this review has several limitations, which means that our results should be interpreted with caution. We only included studies that fit our inclusion criteria described in Section 2.2. Several important studies were excluded due to variety of reasons. These include: Ward et al. only reported the prevalence of vitamin B12 deficiency, but did not show the mean or standard deviation of serum concentrations of vitamin B12 [38]. We excluded this study based on our inclusion criteria (3). Jayaprakash et al. reported their data without a healthy control group, and some of the patients they included had been receiving vitamin B12 supplementation [39]. We excluded this study based on our inclusion criteria (2) and (4). The data included in the analysis were from observational studies; thus, potentially confounding factors may have been included in the baseline characteristics of the selected population. The serum concentrations of folate and vitamin B12 could be affected by ethnicity, gender and complications. Moreover, case-controlled studies and cross-sectional studies lack sufficient proof of the causal link between serum concentrations of folic acid and vitamin B12 and IBD status. Also, observational studies alone to prove cause–effect relationship between certain nutrition factors and certain diseases are very difficult unless the study is as large as the China–Cornell–Oxford Project in the late 20th century [40]. Future prospective studies are needed to identify the causal factors linking serum folate and vitamin B12 concentrations and IBD. 

## 5. Conclusions

In conclusion, the serum folate concentrations in patients with IBD were lower than those in healthy controls, and low serum concentrations of folic acid may be an important risk factor for IBD patients. In addition, the serum vitamin B12 concentrations were lower in Asian patients. We recommend routine screening of patients with IBD for vitamin B12 and folate deficiency to determine whether supplements of folic acid or vitamin B12 should be administered to ensure the nutritional status of IBD patients and to improve the health of patients.

## Figures and Tables

**Table 1:** Studies showing the serum folate and vitamin B12 concentrations in IBD patients and controls.

Author	Region	Score	Year	*n*		Assay Method	Age	Gender (Male/Female)		IBD/CD/UC (Mean ± SD)		Controls (Mean ± SD)		*p*	
				IBD/CD/UC	C		IBD/CD/UC/C	IBD/CD/UC	C	FA (ng/mL)	B12 (pg/mL)	FA (ng/mL)	B12 (pg/mL)	FA	B12
Jiang et al. [22]	China	8	2010	252	654	chemiluminescence	-/-/45 ± 14/46 ± 17	147/105	279/374	4.97 ± 2.73 *	437.53 ± 174.12 *	6.74 ± 3.41	572.77 ± 175.33	<0.001	<0.001
Lambert et al. [23]	France	6	1996	21	20	radioimmunoassay	-	11/10	8/12	2.8 ± 2.8 ^#^	190 ± 146 ^#^	4.55 ± 3.26	207 ± 75	NS	NS
Kuroki et al. [24]	Japan	8	1993	24	24	radioimmunoassay	-/26.4 ± 12.4/-/27.0 ± 4.2	17/7	14/10		641 ± 186 ^#^		682 ± 488	<0.001	
Fernández-Miranda et al. [25]	Spain	7	2005	52	186	radioimmunoassay	41.7 ± 11.9/-/-/41.9 ± 10.1	23/29	71/115	7.6 ± 4.1	499 ± 287	8.9 ± 3.7	603 ± 231	<0.05	<0.05
Erzin et al. [26]	Turkey	7	2008	105	85	ELISA	38.69 ± 12.13/-/-/37.61 ± 10.05	-	-	3.72 ± 1.44	600.14 ± 145.30	4.96 ± 1.19	700.32 ± 141.58	<0.001	
Alkhouri et al. [27]	U.S.	8	2013	61	61	-	12.3 ± 3.9/12.1 ± 4.1/12.3 ± 3.5/12.1 ± 3.6	40/21	30/31	20.1 ± 6.5	775 ± 441	20.4 ± 5.5	727 ± 346		
										20.3 ± 7 ^#^	781 ± 372 ^#^				
										20.9 ± 5.9 *	906 ± 669 *				
Yakut et al. [15]	Turkey	6	2010	138	53	chemiluminescence	-	64/74	19/34	7.7 ± 5.3 ^#^	281 ± 166 ^#^	9.9 ± 3.3	342 ± 179	NS	NS
										8.6 ± 8.3 *	348 ± 218 *				
Akbulut et al. [28]	Turkey	9	2010	55	45	chemiluminescence	-/-/47.4 ± 13.80/46.4 ± 13.89	38/17	31/14	5.1 ± 2.19 *	250.4 ± 82.49 *	6.3 ± 0.87	327 ± 73.9	<0.001	<0.001
Kallel et al. [29]	Tunisia	6	2011	89	103	specific immunochemical methods	35.3 ± 12.6/-/-/36.5 ± 9.26	47/42	50/53	8.54 ± 3.04	295 ± 180	8.1 ± 3.11	378 ± 170	NS	<0.001
Geerling et al. [30]	Netherlands	7	2000	69	69	radioimmunoassay	-/30.1 ± 10.2/37.8 ± 14.7/35.4 ± 13.7	33/36	23/46	4.72 ± 4.02 ^#^	304.96 ± 82.27 ^#^	5.47 ± 2.47 *	357.82 ± 121.58 *	NS	0.05
										5.03 ± 3.80 *	364.59 ± 119.14 *	5.78 ± 2.96 ^#^	365.95 ± 119.54 ^#^		
Chen et al. [31]	China	6	2011	112	110	ELISA	-/-/39.4 ± 11.7/40.3 ± 10.8	58/54	56/54	3.37 ± 0.86 *	147.25 ± 43.67 *	4.03 ± 0.54	152.67 ± 45.17	0.005	0.004
Koutroubakis et al. [16]	Greece	6	2000	108	74	IMx assay	-	66/42	-	6.34 ± 3.05 ^#^	666.5 ± 366.8 ^#^	8.78 ± 3.07	377.5 ± 155.6	<0.05	<0.05
										7.02 ± 3.13 *	478.8 ± 257.7 *				

* Folate and Vitamin B12 levels reported in ulcerative colitis. ^#^ Folate and Vitamin B12 levels reported in Crohn’s disease. - Not applicable. P: patients vs. controls; IBD: Inflammatory Bowel Disease; CD: Crohn’s Disease; UC: Ulcerative Colitis; C: Controls; FA: Folate Acid; B12: Vitamin B12. SD: standard deviation. IMx assay: a multi-functional immune assay system developed by Abbott Diagnostics, Abbott Park, IL.

**Table 2:** Results of the folate regression analysis.

Variables	*n*	*I*^2^	Adj *R*^2^	exp(b)	Std. Err.	*t*	*p*	95% CI
Testing method	10	0.00%	100.00%	0.81	0.10	−1.60	0.154 *	0.61, 1.10
				0.55	0.08	−4.00	0.005 ^#^	0.38, 0.78
				0.73	0.08	−2.95	0.021 ^&^	0.56, 0.94
Year of publication	14	76.64%	−8.34%	1.007	0.02	0.35	0.729	0.97, 1.05
Sample size	14	76.12%	−11.49%	1.00	0.00	−0.35	0.729	0.99, 1.00
Quality of studies	14	76.51%	−10.63%	0.99	0.10	−0.05	0.959	0.79, 1.25
Average age of patients	9	80.56%	18.27%	0.98	0.01	−1.58	0.159	0.95, 1.01

*n*: number; *I*^2^: percentage of total variation across studies; Adj *R*^2^: adjusted *R*^2^, the proportion of between-study variance; exp(b): the exponentiation of the B coefficient; Std. Err: standard error; *t*: *t* statistic, the coefficient divided by its standard error; *p*: an independent variable would be significant (<0.05) or not significant (≥0.05) in the model; CI: confidence interval; * chemiluminescence; ^#^ radioassay; ^&^ ELISA.

**Table 3:** Results of the vitamin B12 regression analysis.

Variables	*n*	*I*^2^	Adj *R*^2^	exp(b)	Std. Err.	*t*	*p*	95% CI
Testing method	11	73.32%	−1.84%	0.86	0.27	−0.47	0.654 *	0.43, 1.76
				1.22	0.37	0.66	0.530 ^#^	0.60, 2.47
				0.67	0.16	−1.64	0.139 ^&^	0.38, 1.18
Year of publication	15	84.87%	16.62%	0.96	0.02	−1.74	0.105	0.92, 1.01
Sample size	15	81.47%	12.78%	1.00	0.00	−1.58	0.139	0.99, 1.00
Quality of studies	15	82.38%	20.13%	0.78	0.10	−1.92	0.078	0.60, 1.03
Average age of patients	10	65.85%	55.42%	0.97	0.01	−2.73	0.026	0.95, 1.00

*n*: number; *I*^2^: percentage of total variation across studies; Adj *R*^2^: adjusted *R*^2^, the proportion of between-study variance; exp(b): the exponentiation of the B coefficient; Std. Err: standard error; *t*: *t* statistic, the coefficient divided by its standard error; *p*: an independent variable would be significant (<0.05) or not significant (≥0.05) in the model; CI: confidence interval; * chemiluminescence; ^#^ radioassay; ^&^ ELISA.
